# PVA Nanofibers as an Insoluble pH Sensor

**DOI:** 10.3390/polym15234480

**Published:** 2023-11-21

**Authors:** David Mínguez-García, Ignacio Montava, Marilés Bonet-Aracil, Jaime Gisbert-Payá, Pablo Díaz-García

**Affiliations:** Departamento de Ingeniería Textil y Papelera, Universitat Politècnica de València, Plaza Ferrándiz y Carbonell nº1, 03801 Alcoy, Spain

**Keywords:** turmeric, citric acid, cross-linking

## Abstract

Turmeric has been widely studied as a color indicator for pH variations due to its halochromic properties. It has been tested in solution or included in some polymeric matrices. Some studies have demonstrated that its change in color is due to the tautomeric species of curcumin, and this property can be observed even if turmeric is assimilated in a film or nanofiber. Chitosan/polyethylene oxide (PEO) polymers have been tested in previous studies. Polyvinyl alcohol (PVA) nanofibers are used as potential carriers of drugs once they are insolubilized. The aim of this work is to cross-link PVA with citric acid (CA) to insolubilize the nanofibers and determine the effect on turmeric’s halochromic properties. The nanofibers were treated with a sodium hydroxide (NaOH) solution, and a chromatic study was undertaken to determine color change. The change in color was assessed by eye (subjective) and by spectroscopy (objective). The nanofibers were characterized, in addition to the colorimetric study, by Fourier transform infrared (FTIR) spectroscopy and scanning electron microscopy (SEM) as well. The results demonstrate how thermal treatment induces cross-linking between the nanofibers, allowing them to keep their shape once the NaOH solution is applied to them. The opposite effect (solubilization) can be observed for non-cross-linked (NCL) samples. Although the final color varied, the cross-linked (CL) nanofibers’ halochromic behavior was maintained. It was demonstrated that during cross-linking, ester groups are formed from the free carboxyl group in the cross-linked CA and the ketones present in the curcumin under acid conditions. So, CA acts as an acid catalyst to bond turmeric to the cross-linked PVA nanofibers.

## 1. Introduction

The substantial progress and development that society has undergone in recent decades has brought about a significant change in many people’s lifestyles. Nowadays, human beings are purchasing smaller, safer, cheaper, and more rapidly produced products. Nanotechnology is a technique capable of producing and manipulating matter at the nanometer scale to create new structures, materials, and products [1].

Nanotechnology has been able to demonstrate that materials at the nanometer scale can offer different characteristics than when they are at a larger scale. For example, ceramic materials have high brittleness except when they are present as nanoparticles, when they can be malleable. When working at the nanometer scale, elements such as copper can form substances that turn from opaque to transparent, and gold can change from insoluble to soluble [1].

The advance of nanotechnology has driven development and innovation in the textile industry, enabling the manufacture of smart and multifunctional textiles with applications in various areas of health, pharmaceuticals, fashion, sports, transportation, protection, and high-performance products. Smart textiles are able to detect an external stimulus, whether mechanical, thermal, chemical, magnetic, electrical, optical, or physiological, and respond to it. Functional fabrics are designed to exhibit functions in addition to their aesthetic properties [2].

The modification of textiles at the nanoscale allows the implementation of new functionalities or improvements in their performance without overshadowing their initial characteristics of lightness, flexibility, comfort, and aesthetics. The new functions provided can include hydrophobicity [3,4,5], oleophobicity [6,7], antistatic properties [8,9], resistance to wrinkling [10], improved resistance to mechanical stress [11,12,13], protection against ultraviolet radiation [14,15], antibacterial capacity [14,16,17,18], and complex developments such as flexible bioelectronics [19] among other characteristics.

In the textile industry, nanotechnology has also made its presence felt in the production of fibers. One of the main objectives of chemical spinning is to produce fibers with the smallest possible cross-section. Based on this objective, electrospinning has emerged as one of the most promising technologies capable of producing fibers with a sub-micrometer or even nanometer cross-section. Moreover, electrospinning is also a simple and versatile technique capable of producing nanofibers with a multitude of morphologies using highly diverse materials, from polymeric to ceramic materials, for example. The technique is able to offer exceptional characteristics, such as a high surface-to-volume ratio, an ultrafine fibrous structure connected to itself at a multitude of points, and high porosity and lightness [2,19,20,21].

Another feature of the electrospinning technique is the possibility of adding different compounds to nanofibers, both superficially adhered and encapsulated inside them. Due to the simplicity and versatility of the nanofiber morphologies that can be obtained, this technology has been implemented in many industrial sectors, including self-cleaning textiles with applications in medical devices and protective clothing [2,22], agrotextiles with living matter inside the nanofibers [23], air filtration products, water treatment filters, catalysis processes, enzyme immobilization, and solar cells. Nanofibers are used in a wide variety of products in the biomedical sector, with applications including cellular tissue repair and regeneration, wound healing and scarring, musculoskeletal tissue regeneration, tendon/ligament regeneration, cancer diagnosis and treatment, and implant coating [2].

However, most of the research carried out to date has focused on the technical aspects of nanofibers, and their aesthetic side has been completely overshadowed by their excellent and diverse qualities that provide great value across many different sectors. However, their aesthetic aspect can also provide technical value; several authors have used different halochromic compounds encapsulated inside electrospun nanofibers as sensors to detect pH changes in the environment. The incorporation of halochromic compounds into nanofibers involves a dyeing process since the principle of halochromism is based on a color change in the compound as a function of the pH in which it is found.

Agarwal et al. made electrospun PA 6 nanofibers that could encapsulate different halochromic dyes, such as Phenol Red, Methyl Red, Bromothymol Blue, phenolphthalein, and Bromocresol Green, with the aim of being able to detect color changes from pH 1 to pH 10, in a study that also determined the effect of the compound on fiber morphology [24]. Van der Schueren et al. used the halochromic dyes Bromocresol Purple and Brilliant Yellow to dye PA 6.6. nanofibers, and they concluded that the incorporation of the pH indicator did not influence the average diameter of the fibers; however, they did find an influence on the formation of droplets during the process when the dye had not been properly dissolved. Nanofibers incorporating both dyes demonstrated good characteristics as pH indicators [25]. Tripathy et al. developed PA 6 nanofibers dyed with the halochromic dyes Phenol Red, Bromocresol Purple, and phenolphthalein to determine the pH change of milk as a function of its purity [26]. In the medical field, nanofibrous biosensors have been designed with the dye Bromothymol Blue for the rapid and visual detection of wound infection [27].

**Figure 1:**
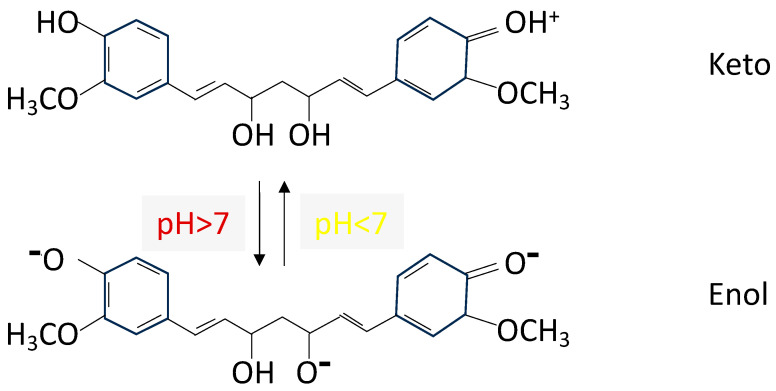
Curcumin structure depending on pH.

Naturally occurring dye compounds that offer halochromic characteristics have been used by other authors due to their chemical composition, in addition to being renewable, non-toxic, non-polluting, safe and biocompatible. Devarayan and Kim prepared electrospun cellulose nanofibers containing a natural pigment extracted from red cabbage; the pigment’s anthocyanin content results in a color change across a pH range of 1 to 14. They suggest a possible application in medical monitoring of alcohol intake levels via a saliva test [28]. Maftoonazad and Ramaswamy also employed red cabbage pigment along with polyvinyl alcohol for the production of electrospun nanofibrous mats for monitoring pH changes in fresh date packaging [29]. Extensive study has also been performed on curcumin, a natural lipophilic pigment found in turmeric and extracted from *Curcuma longa*, which possesses the ability to change its color due to its diketone groups being converted to the keto-enol form when exposed to alkaline conditions [30] (see Figure 1). Researchers have fabricated various nanofibers with encapsulated turmeric for visual monitoring of the pH of fresh food packaging [30,31,32]. 

The present research proposes polyvinyl alcohol (PVA) nanofibers incorporating turmeric as a coloring agent in the polymeric solution for future application as a pH sensor in the field of air filtration. PVA is characterized by being a hydrophilic, non-toxic, biodegradable, biocompatible, semicrystalline polymer, qualities that allow it to be used in a wide variety of sectors. However, it has low water stability, which limits its applicability; as a result, PVA is often treated to ensure prolonged stability and improve its performance [33]. The methods studied to improve its stability in water have included crystallization by freezing and thawing, irradiation, acid-catalyzed dehydration, heat treatments, radical production, and treatment with formaldehyde or glutaraldehyde [34]. However, some of these methods are toxic or expensive; therefore, several authors have used citric acid (CA) as a green method for PVA cross-linking. CA is a non-toxic polycarboxylic acid that can react with the hydroxyl groups of PVA chains and give rise to intermolecular and intramolecular ester bonds between them. The cross-linking reaction between PVA and CA occurs upon heating to high temperatures, from 130 to 220 °C, for long periods of time, depending on the temperature [33,35,36].

The objective of this study was to determine if cross-linking between PVA and CA influences the ability of the halochromic sensor to detect pH changes and generate color changes in turmeric. The possibility of creating non-water-soluble halochromic PVA nanofibers will increase PVA’s field of application. Due to the possible presence of suspended substances in the turmeric dispersion, the dispersion used in the study was filtered to avoid alterations in the electrospinning process. Another unfiltered dispersion was used to evaluate whether there are differences after filtration in terms of coloration and detection of pH change.

## 2. Materials and Methods

### 2.1. Materials

The nanofibers were composed of polyvinyl alcohol (PVA) Mw 61,000 g/mol with a degree of hydrolysis of 98.0–98.8 mol% at 99% ± 0.8 purity, purchased from Sigma-Aldrich (Akralab, Alicante, Spain); turmeric, purchased from a local herbalist; and citric acid (CA) at 99% purity purchased from Sigma Aldrich. The PVA with CA and turmeric dispersion was prepared in distilled water.

The vertical metal collector of the electrospinning system was covered with wax paper supplied by Bosque Verde (Tarragona, Spain). The halochromic effect was evaluated using a 10 g/L solution of sodium hydroxide (NaOH) supplied by Sigma Aldrich, which has a pH value of 11–12.

### 2.2. Methods

To produce the dispersion to be electrospun, the turmeric was first dispersed at 5 g/L in distilled water with electromagnetic stirring for 2 h. After this time, half of the prepared dispersion was filtered using a sintered glass fiber disc with a nominal pore diameter of 1.2 µm. Subsequently, in both turmeric dispersions (filtered and unfiltered). PVA was added at 9% *w*/*v* and CA at 10% *w*/*v* with respect to the weight of PVA and stirred with an electromagnetic stirrer at 80 °C until complete dissolution of the PVA.

To characterize the viscosity of the dispersions, a FUNGILAB Visco Elite R viscometer from Instrumentación Analítica (Barcelona, Spain) was used with an R3 spindle and a stirring speed of 200 RPM. The electrical conductivity was measured at a temperature of approximately 22 °C in both solutions with a Crison Basic 30 Conductimeter (Hach Lange Spain. S.L.U., L’ Hospitalet de Llobregat, Spain). Surface tension was measured with a KRÜSS K9 tensiometer (Krüss, Hamburg, Germany). For pH measurement, a GLP 22 pH meter from CRISON (CRISON, Barcelona, Spain) was used.

The electrospinning process was carried out using a Spinbox instrument from Bioinicia (Bioinicia, Paterna, Spain). The instrument’s vertical metallic collector was covered with white waxed paper, which the nanofibers were deposited onto to facilitate their handling. A 16-gauge capillary was chosen for the extrusion of the solutions to avoid clogging by the non-dispersed compounds. The extrusion had the following characteristics: a rate of 0.8 mL/h, an applied voltage of 19 kV, a distance between the extruder electrode and the collector of 12 cm, and a process duration of 30 and 120 min. The temperature of the environment where the experiment was performed was 25 °C, with a relative humidity of 53%.

To cross-link the nanofiber veils, a TCF 120 oven from ARGO LAB (ARGO LAB, Carpi, Italy) was used at a temperature of 190 °C for 10 min.

The first characterization of the nanofibrous veils was undertaken with the naked eye by 5 volunteers to determine whether there was coloration in the electrospun nanofibers and to determine which of them had a greater or lesser color tonality.

The color of the nanofiber veils was determined by using a MINOLITA CM-3600d (Konica Minolta Business Solutins Spain, Barcelona, Spain) reflection spectrophotometer. UV energy was included. The measurements were made with the CIE-Lab 10° standard observer and the standard illuminant D65 [37]; the values for L, a, and b for the evaluation of color and the total color difference (ΔE) were calculated using Equation (1).

(1) $$\mathrm{DE}=\sqrt{{\left(L-L^{*}\right)}^{2}+{\left(a-a^{*}\right)}^{2}+{\left(b-b^{*}\right)}^{2}b^{2}}$$

As the electrospun samples were circular in shape (see Figure 1), the color measurement was conducted in the center of the circle.

The samples were examined by electron microscopy using a field emission scanning electron microscope FESEM ULTRA 55(Carl Zeiss, Jena, Germany). Each sample was placed on a surface and covered with a layer of gold and palladium using a sputter coater in order to make the samples conductive. The samples were analyzed with the appropriate magnification and with an acceleration voltage of 2 kV.

Fourier transform infrared (FTIR) spectra were recorded in order to characterize each fabric’s surface. A JASCO FT/IR-4700 type A spectrometer (Jasco Spain, Madrid, Spain) with an ATR accessory was used to record 16 spectra with a 4 cm^−1^ resolution.

## 3. Results

### 3.1. Solution Characterization

As a starting basis for sample characterization, a single polyvinyl alcohol dilution was tested against filtered (PVA_F) and unfiltered (PVA_NF) turmeric dispersions.

The dispersion was filtered through a sintered glass fiber disc to remove any turmeric particles larger than 1.2 µm that could interfere with the electrospinning process by obstructing the capillary exit or hindering the formation of the Taylor cone. Table 1 shows the characterization parameters for the PVA preparations for electrospinning.

The characterization demonstrated the changes that occurred in the PVA starting solution when citric acid and turmeric were added. It can be seen how the presence of these compounds increased the viscosity up to 192.70 cP in the case of the PVA_NF dispersion; however, when the dispersion was filtered, it had a value slightly lower than that of the starting solution, 129.02 cP. The large increase in the conductivity parameter also observed was due to the addition mainly of CA, since it dissociates into hydrogen ions and citrate ions. The surface tension decreased only slightly in both dispersions with respect to the PVA solution. However, there was a noticeable reduction in pH in the dispersions due to their CA content with respect to the neutral pH from the initial submission.

### 3.2. Nanofiber Veil Characterization

#### 3.2.1. Visual Color Test

**Figure 2:**
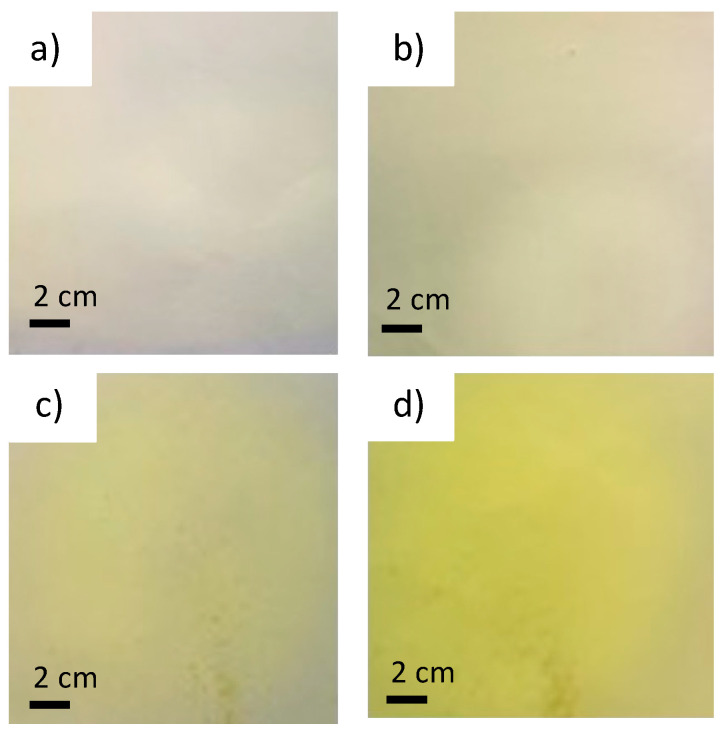
Visual observation of electrospun nanofibers. (**a**) PVA_F_30; (**b**) PVA_F_120; (**c**) PVA_NF_30; (**d**) PVA_NF_120.

In order to prove that, in addition to the filtration of the solution, there is also a difference in the color of the nanofiber mat due to the electrospinning time, two electrospinning process times were employed, 30 and 120 min; thus, four samples were obtained: the samples from the filtered (F) solution electrospun for 30 min (PVA_F_30) and 120 min (PVA_F_120) and the nanofibers produced from the non-filtered (NF) solution electrospun for 30 min (PVA_NF_30) and 120 min (PVA_NF_120). The visual appearance of the samples can be seen in Figure 2. As can be observed, the PLA without turmeric is white for both the 30 and 120 min samples (Figure 2a and 2b, respectively). As can also be seen, turmeric inclusion gives a yellow color for both the 30 min and 120 min samples (Figure 2c and 2d, respectively). The SEM results for the samples are show in Section 3.4.

The visual test was carried out by five volunteers. They were presented with the four samples prior to cross-linking and were asked to rank, from highest to lowest, which sample had the yellowest coloration following the incorporation of turmeric as a dye. Figure 2 shows the samples presented to the volunteers. The samples were presented to the individuals unlabeled; the volunteers simply had to order them from left to right, from highest appreciation of yellowish hue to lowest.

All the volunteers, without knowing the order decided by the other volunteers or the conditions for the electrospinning, ordered the samples in the same way, as shown in Table 2.

#### 3.2.2. Reflectance Spectroscopy

##### Initial Samples

Reflectance spectroscopy was carried out to determine the coloration of each sample accurately and quantitatively. Measurements of the chromatic coordinates of each sample were taken at four different positions.

Table 3 shows the CIE L*a*b* values obtained from the electrospun samples. Waxed paper was used as the standard sample because it was used as the collector substrate on which the nanofibers were collected and was kept during the analysis to facilitate the handling of the nanofibers.

Table 3 shows that all the samples present a difference with respect to the standard sample in their DE*ab values. It can be seen that the nanofiber samples produced from the unfiltered PVA solution have higher color difference values, with the 30 min electrospun sample having a value of 21.23 DE*ab and the 120 min sample having a value of 25.40 DE*ab. Similarly, it can be observed that all the samples have very similar brightness values that are close to the value obtained for the collector wax paper; however, it is noteworthy that the samples electrospun for a longer time, PVA_NF_120 and PVA_F_120, have higher brightness values, 96.42 and 97.60 L*, respectively.

##### Cross-Linked Samples

Due to the high solubility of PVA in water, a cross-linking process must be carried out to facilitate the handling and applicability of the electrospun veils. After cross-linking, a change in the color shade of the samples, including the waxed paper, can be observed. The CIE L*a*b* color coordinates of the electrospun samples after cross-linking are shown in Table 4.

The waxed paper was also subjected to the same cross-linking process to see if it undergoes alterations on its own.

The results obtained from the measurements of the cross-linked nanofibers show chromatic differences in relation to the original samples. The values obtained for the color differences with respect to the base surface on which the solutions were electrospun, waxed paper, are shown. The brightness values were hardly altered after cross-linking.

The value of 15.37 DE*ab obtained for the cross-linked waxed paper shows a notable color difference with respect to the original sample (0 DE*ab). The cross-linking of the unfiltered samples, PVA_NF_30_CL and PVA_NF_120_CL, hardly alters the initial coloration as the DE *ab values are similar to those before cross-linking. On the other hand, the color difference for the PVA_F_30_CL and PVA_F_120_CL nanofibers has increased with respect to the original samples, with values of 13.75 DE*ab and 14.55 DE*ab, respectively, obtained after cross-linking.

In all cases, the samples show high luminosity values. It can be seen that the samples electrospun for 120 min both present a higher luminosity than the samples that have been electrospun using the same solution but for 30 min.

### 3.3. Halochromic Study

After the halochromic test was performed on the electrospun samples, both thermally cross-linked and non-cross-linked, new chromatic values were obtained.

Table 5 shows the CIE L*a*b* values obtained from the electrospun samples after the halochromic test was performed on the nanofibers. Waxed paper was still used as the standard sample.

**Figure 3:**
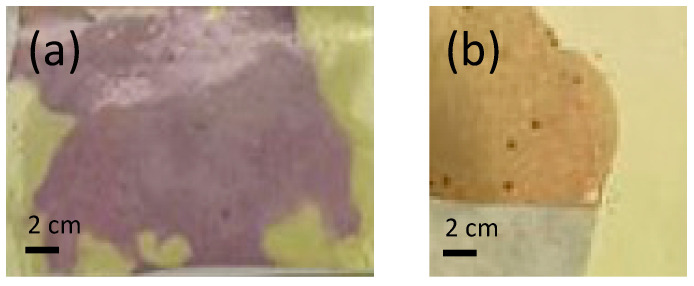
Halochromic behavior for PVA nanofibers: (**a**) non-cross-linked; (**b**) cross-linked.

Figure 2 clearly shows the color change resulting from performing the halochromic test. Figure 3a shows the results when the NaOH solution is in contact with the sample with the unfiltered turmeric solution electrospun for 120 min (PVA_NF_120). 

The halochromic test was also performed on the electrospun samples that were cross-linked to obtain an insoluble PVA (Figure 3b). The values obtained are shown in Table 6.

### 3.4. SEM

**Figure 4:**
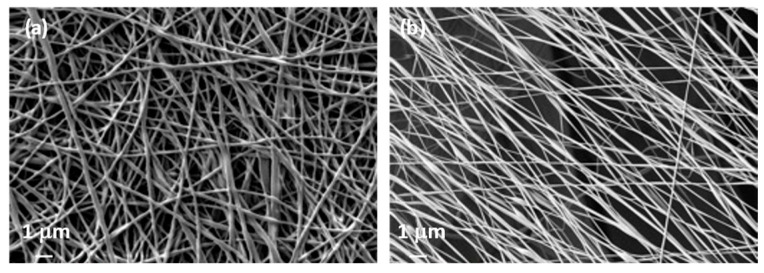
PVA nanofibers. (**a**) Irregular nanofibers, solvent not evaporated; (**b**) regular nanofibers, solvent correctly evaporated (PVA non-cross-linked 30 min).

The conditions used for PVA electrospinning were defined based on the microscopy analysis to ensure that no bed or solvent presence was visible. Figure 4a shows PVA nanofibers that have no bed but arrive at the collector without the complete evaporation of the solvent. In contrast, Figure 4b shows clearly defined nanofibers with no solvent present. Thus, these were the conditions selected when conducting this study and the ones reported in Section 2.2.

**Figure 5:**
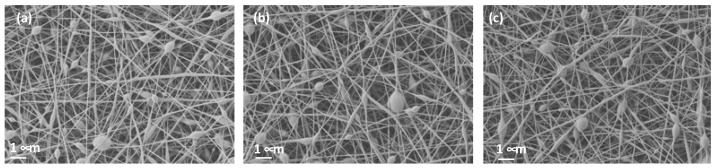
Effect of filtering and cross-linking on nanofibers. (**a**) Non-filtered non-cross-linked (NF-NCL); (**b**) filtered and non-cross-linked (F-NCL); (**c**) filtered and cross-linked (F-CL).

When the citric acid and turmeric were included in the PVA solution, the parameters varied, and although, apparently, a Taylor cone was created, the nanofibers generated beds, which indicated that the conditions should be changed (Figure 5). However, no changes were made to the electrospinning conditions as the solution parameters for conductivity and viscosity were considerably increased by the addition of turmeric and CA (Figure 5a), but they slightly decreased when filtered (Figure 5b), and in both cases, the Taylor cone was consistent. Small changes in the parameter resulted in no nanofibers being formed. Figure 5c shows the nanofibers’ appearance once the nanofibers had been cross-linked (CL) by thermal treatment.

**Figure 6:**
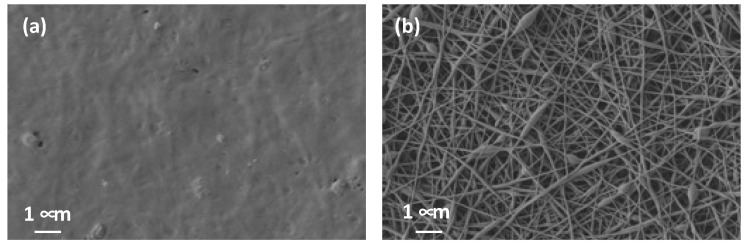
Effect of NaOH solution (H) on nanofibers. (**a**) Filtered and non-cross-linked (F-NCL_H); (**b**) filtered and cross-linked (F-CL_H).

Once the nanofiber veils were obtained, a drop of NaOH solution was placed on the surface of the nanofibers and left to dry. This was done on both cross-linked (CL) and non-cross-linked (NCL) nanofibers. The veils were subsequently analyzed by SEM in order to determine the effect. As can be seen in Figure 6, the NCL nanofibers completely dissolved, whereas the ones that had been thermally treated to induce cross-linking between the PVA and CA still showed a nanofiber net.

### 3.5. FTIR

**Figure 7:**
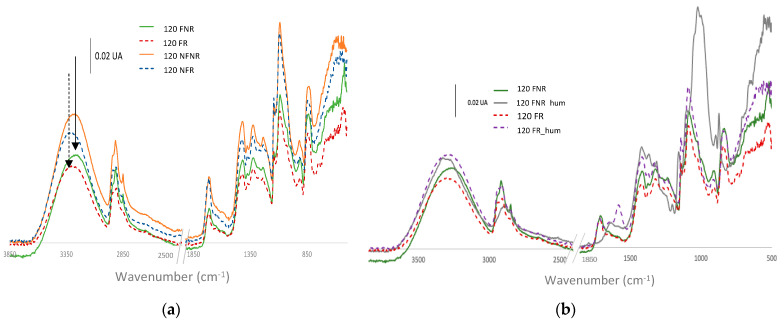
ATR−FTIR spectra from PVA nanofibers with turmeric and citric acid solution. (**a**) Comparison of filtered (F) and non-filtered samples (NF) that were cross-linked (CL) or non-cross-linked (NCL); (**b**) comparison of halochromic behavior for cross-linked (CL) and non-cross-linked (NCL) samples as obtained or following alkaline treatment (H).

The FTIR spectra for the different samples were analyzed; the most important findings are shown in Figure 7. The influence of CA cross-linking can be appreciated when the spectra are compared with samples without the thermal treatment (NCL). The OH stretching band is altered due to the cross-linking. As can be seen in Figure 7a, the peak is centered around 3260 cm^−1^ for the non-cross-linked (NCL) samples and represented by lines, whereas the samples with thermal treatment show a shift towards a higher wavelength centered around 3306 cm^−1^. When the effect of filtration is compared (filtered (F) versus non-filtered (NF)), it can be clearly seen that there is a slight difference, mainly observed in the region 800–500 cm^−1^, which corresponds to the ring and OH bands [38]. For the non-filtered samples, the peaks in that region were lower, and this may be due to the presence of agglomerated particles that were retained by the filter.

## 4. Discussion

### 4.1. Effect of Solution Filtration and Electrospinning Time on Nanofiber Color

**Figure 8:**
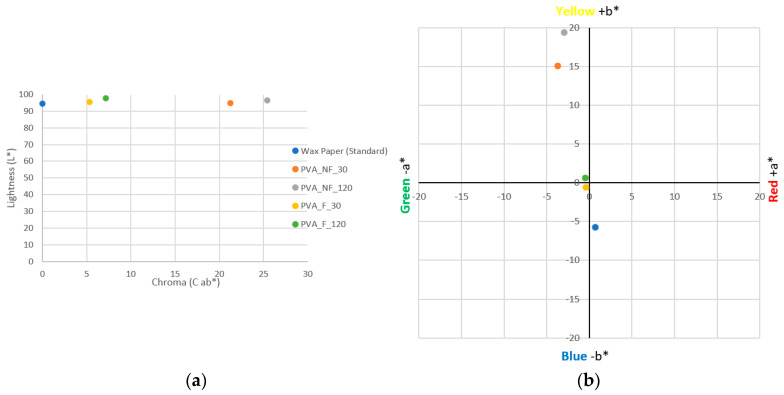
Colorimetry results for the samples. (**a**) Sample brightness results. (**b**) Chromatic coordinates; blue circle: wax paper (standard); orange circle: PVA_NF_30 nanofibers; grey circle: PVA_NF_120 nanofibers; yellow circle: PVA_F_30 nanofibers; green circle: PVA_F_120 nanofibers.

Figure 8 shows the coordinates on the CIE L*a*b* space of the electrospun samples and the collector wax paper of the nanofibers. The results show a brightness value for the collector wax paper of 94.45 L*; however, the rest of the samples have values higher than this, indicating a higher brightness in all the electrospun samples (Figure 8a).

For the electrospinning samples, the lowest value obtained is for the PVA_NF_30 sample, with a value of 94.63 L*, while the filtered sample with the same electrospinning process time, i.e., the PVA_F_30 sample, has a slightly higher brightness, with a value of 95.38 L*. The samples electrospun for a longer time, 120 min, have higher brightness values. The sample electrospun from the unfiltered solution (PVA_NF_120) has a value of 96.42 L*, while the filtered sample, PVA_F_120, has the highest brightness value, 97.60 L*.

Therefore, with respect to the electrospun samples, it is possible to state that the filtration of the turmeric solution increases the luminosity of the nanofibrous mats, in addition to it being possible to observe that a longer electrospinning time, i.e., a higher deposition of nanofibers, is also a parameter that increases the luminosity value.

Regarding the color differences (DE*ab) (Figure 8b) with respect to the standard sample, it can be observed that the unfiltered samples, PVA_NF_30 and PVA_NF_120, have higher values than the filtered samples, 21.23 and 25.40 DE *ab, respectively. The nanofibrous sample obtained from the turmeric solution filtered and electrospun for 30 min (PVA _F_30) has a value of 5.31 DE*ab, a lower value than the sample electrospun for 120 min from the same solution (PVA _F_120), which has a value of 7.16 DE *ab.

The results demonstrate in both cases the higher color difference, i.e., the higher presence of color, in the nanofibers when electrospinning takes place for longer process times, 120 min in this case. They also show a notable difference in coloration depending on whether the PVA solution has been filtered or not, with the presence of darker areas observed when the solutions have not been filtered (Figure 3b).

In Figure 8b, the chromatic coordinates measured on the samples show that the waxed paper has a slight and barely noticeable blue coloration, with a value of −5.70 b*. The coordinates of the PVA_F_30 nanofibers increase on the Y-axis with respect to the standard sample, with a value of −0.59 b*; however, they remain very close to the center of both axes, so no color predominates on the nanofiber surface. The sample filtered and electrospun for 120 min (PVA_F_120) shows a slight increase in coloration with respect to the previous sample since it has chromatic coordinates of −0.48 a* and 0.62 b*, which is explained by a slightly higher coloration in the nanofibers.

The electrospun nanofibers from the unfiltered turmeric solution have very different chromatic coordinates than those obtained from the filtered solution. The PVA_NF_30 nanofibers have coordinates of −3.72 a* and 15.07 b*, which reflect a noticeable yellow coloration due to their high value on the Y-axis. The PVA_NF_120 sample has coordinates of −2.92 a* and 19.36 b*, where the value of +b*, responsible for the yellow coloration, is higher than that for the rest of the samples, so this sample is the one with the greatest yellow coloration.

The chromatic coordinates have demonstrated that a greater coloration is obtained for the nanofibers when the turmeric solution is not filtered, in addition to the fact that a more intense color is obtained with longer electrospinning times. These results, as could be expected, agree with the results obtained from the volunteers’ observations (Table 2).

The filtration of the solution did not make any difference to the appearance of the nanofibers; while the image for the non-filtered samples has some areas with a higher intensity of color (see Figure 3), the color was the same for the filtered (F) samples compared to the non-filtered (NF) samples.

### 4.2. Effect of Cross-Linking on Nanofiber Color

From the data shown in Table 3 and Table 4, it can be determined whether the thermal cross-linking process has an influence on the color present in the electrospun nanofibers.

**Figure 9:**
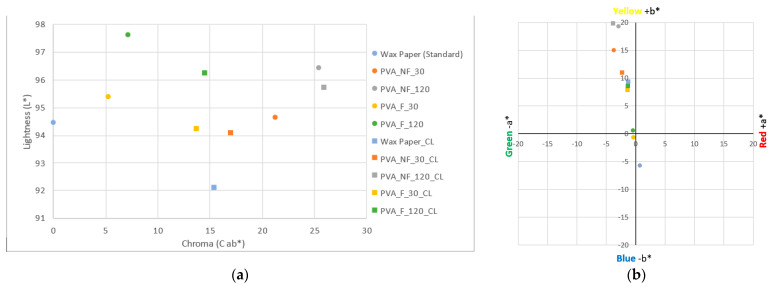
Colorimetry results for the samples. (**a**) Sample brightness results. (**b**) Chromatic coordinates; blue circle: wax paper (standard); orange circle: PVA_NF_30 nanofibers; grey circle: PVA_NF_120 nanofibers; yellow circle: PVA_F_30 nanofibers; green circle: PVA_F_120 nanofibers; blue square: cross-linked wax paper; orange square: PVA_NF_30_CL nanofibers; grey square: PVA_NF_120_CL nanofibers; yellow square: PVA_F_30_CL nanofibers; green square: PVA_F_120_CL nanofibers.

Figure 9a represents the values obtained from the original samples versus the cross-linked nanofibrous samples. First of all, it is worth noting the influence of the thermal process on the colorimetry of the waxed paper used as a collecting surface; it can be seen that, after cross-linking, its brightness value has decreased to 92.11 L*, and at the same time it has moved along the X-axis to give a value of 15.37 DE *ab, which shows an increase in coloration with respect to its original color.

The electrospun samples after cross-linking show slightly lower values in terms of brightness, as can be seen in the points plotted in Figure 9a; this could be due to the fact that the heat treatment partially opacifies the light of the samples. Regarding the color differences, the same figure shows how the cross-linking process in the filtered samples, PVA_F_30_CL and PVA_F_120_CL, increases their DE ab values until they are very close to the DE *ab value of the collector wax paper.

From Figure 9b, it can be deduced that after cross-linking, the +b* value of the PVA_NF_120_CL sample has remained stable since it had a value of 19.36 b* before the heat treatment and a value of 19.82 b* afterward. In the case of the PVA_NF_30_CL sample, while the cross-linking increased the chromatic coordinates on the Y-axis for the other samples, these coordinate values slightly decreased in this case.

The +b* value of the heat-treated collector wax paper increased. Initially, it had a cold white shade, and hence its value of −5.70 b* is towards the bluish area of the CIE L*a*b* space. After cross-linking, it had a yellowish hue, as indicated by the 9.37 b* value.

The electrospun samples from the filtered solution (PVA_F_30_CL and PVA_F_120_CL) underwent significant color changes after the cross-linking of the polymer. The original samples presented a* and b* values very close to the center of the axes (0.0), thus representing a whitish coloration. However, the location of the points plotted for the cross-linked samples in Figure 9b demonstrates the existence of a yellowish hue in the nanofibers. It is noteworthy that although the b* values of these samples have increased, they remain below the value obtained for the waxed paper; however, this can be explained by the nature of the components. The wax of the paper may have undergone an oxidation process when a high temperature was applied for the cross-linking of the PVA, which resulted in the yellowish coloration.

### 4.3. Halochromic Behavior

The visual analysis performed allowed the determination of the color change in the electrospun nanofibers after the halochromic test was performed on them. The data in Table 3 and Table 5 represent the color differences graphically and quantitatively.

**Figure 10:**
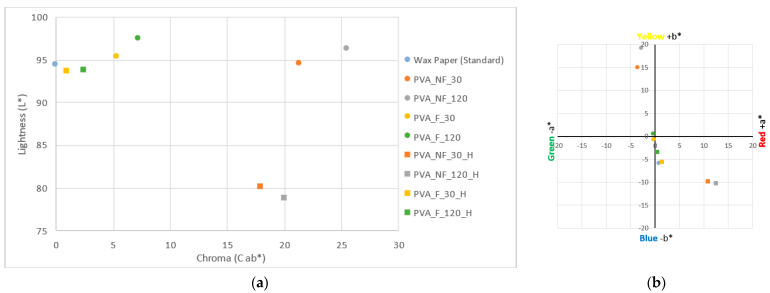
Colorimetry results for the samples. (**a**) Sample brightness results. (**b**) Chromatic coordinates; blue circle: wax paper (standard); orange circle: PVA_NF_30 nanofibers; grey circle: PVA_NF_120 nanofibers; yellow circle: PVA_F_30 nanofibers; green circle: PVA_F_120 nanofibers; blue square: cross-linked wax paper; orange square: PVA_NF_30_H nanofibers; grey square: PVA_NF_120_H nanofibers; yellow square: PVA_F_30_H nanofibers; green square: PVA_F_120_H nanofibers.

**Figure 11:**
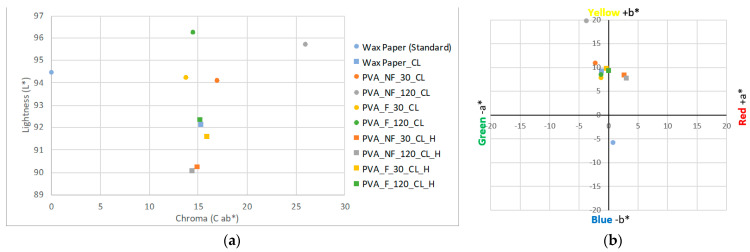
Colorimetry results for the samples. (**a**) Sample brightness results. (**b**) Chromatic coordinates; blue circle: wax paper (standard); orange circle: PVA_NF_30_CL nanofibers; grey circle: PVA_NF_120_CL nanofibers; yellow circle: PVA_F_30_CL nanofibers; green circle: PVA_F_120_CL nanofibers; blue square: cross-linked wax paper; orange square: PVA_NF_30_CL_H nanofibers; grey square: PVA_NF_120_CL_H nanofibers; yellow square: PVA_F_30_CL_H nanofibers; green square: PVA_F_120_CL_H nanofibers.

Firstly, with respect to the brightness values, it can be seen in Figure 10a that the initial samples have higher brightness values in all cases and that these values decrease slightly when the final color is evaluated after the halochromic test. On the other hand, in Figure 11a, it is also noticeable how a relationship is maintained between the initial color difference versus the color difference after the color change; that is, the PVA_F_30 sample represented by the yellow circle has the smallest color difference compared to this sample after being subjected to the halochromic test, as indicated by the yellow square representing the PVA_F_30_H sample.

Therefore, it is possible to affirm that the color shade after the halochromic test is also influenced by the electrospinning process time and the filtration of the solution used. It is noticeable how, in both the filtered and unfiltered solutions, the PVA_NF_120_H and PVA_F_120_H samples, electrospun for 120 min, show a greater color difference after the test than the samples made employing a shorter process time. The unfiltered samples, PVA_NF_30_H and PVA_NF_120_H, show greater color differences with respect to the filtered samples and the collector wax paper.

On the other hand, in Figure 10b, drastic changes in color shades are noticeable. The initial sample PVA_NF_120 is shown in the upper left quadrant of the graph because it has colorimetric values of −2.92 a* and 19.36 b*, which explains its notable yellowish hue. However, the PVA_NF_120_H sample has values of 12.34 a* and −10.13 b*, which places the gray square in the lower right quadrant of the graph, thus giving a final purple hue. The same occurs with the rest of the samples, however, as in the DE *ab color difference graph, the influence of lower coloration is maintained due to the electrospinning time and the filtration of the electrospun solutions.

Therefore, by means of the halochromic test, it has been possible to determine the existence of a color change from yellow to purple for the nanofibers electrospun from the PVA and turmeric solutions. It has also been possible to affirm that the color intensity ratio is maintained with respect to the electrospinning and filtration time of the solutions.

The curcumin in turmeric varies from a keto to an enol tautomer depending on the pH. The enol form is present when it is dissolved in organic solvents or alkaline solutions, whereas the keto form dominates in acid media [39,40]. This halochromic behavior is kept when turmeric is included in some polymeric films or nanofibers [30,31,32]. In this paper, we focus on PVA, and we demonstrate that PVA has the capability to form yellow nanofibers which change their color when in contact with alkaline solutions. This is due to the fact that alkaline solutions make the curcumin lose protons to give a net charge of −3. This shift from the enol to the keto tautomeric form of curcumin is demonstrated by the FTIR spectra (Figure 7b), where a variation can be seen in the OH stretching band (3800–3000 cm^−1^). This is because the number of OH groups decreases when the curcumin moves from the keto to the enol form, and consequently, this is directly related to the color change from yellow (pH ≤ 7) to a reddish hue (pH > 7), as many authors have reported [39,40].

### 4.4. Effect of Cross-Linking on Halochromic Behavior

In the previous sections, the effect of thermal cross-linking on the colorimetric values of the dyed nanofibers has been confirmed; similarly, the color change from yellow to purple after the halochromic test has also been demonstrated. Therefore, in this section, we analyze both of the factors that induce changes; i.e., we evaluate whether the thermal treatment to which the nanofibrous veils are subjected to produce a water-insoluble polymer affects the halochromic capacity generated by the encapsulation of turmeric inside the fibers. The data shown in Table 4 and Table 6 are plotted in Figure 11.

Figure 11a clearly demonstrates that all the cross-linked samples initially show higher brightness values before, rather than after, the halochromic test. This phenomenon may be due to the fact that the nanofibers change from a yellow color with high lightness to a dark brownish shade, as can be seen in Figure 3. As an example of this, the sample PVA_NF_120_CL (represented by a gray circle) had a lightness value of 95.71 L* and a color difference value of 25.95 DE *ab (the highest of all the cross-linked samples) after cross-linking; however, after the halochromic test, it had values of 90.06 L* and 14.44 DE *ab, which also suggests a change in color and luminosity with respect to the collector surface, but to a lesser extent.

Figure 11b shows the colorimetric values of each sample. It can be seen that the values of the sample PVA_NF_120_CL place it in the upper left quadrant, with values −3.83 a* and 19.82 b*, thus demonstrating its yellowish coloration. However, when the halochromic test was performed, the PVA_NF_120_CL_H sample had values of 2.95 a* and 7.87 b*, which placed it in the upper right quadrant, the sample having a brownish color. In the PVA_NF_30_CL_H sample, the same occurs as for the sample electrospun for 120 min; however, its values are lower due to the shorter deposition time during the electrospinning and therefore its shorter coloration time.

The electrospun nanofibers from the filtered turmeric solution hardly underwent a color change when the halochromic test was performed after cross-linking. As shown in Figure 11b, the values obtained after the halochromic color change of the PVA_F_30_CL_H and PVA_F_120_CL_H samples are very close to the values recorded for the same samples after thermal cross-linking; moreover, it is observed that all the values are very close to the color shade of the cross-linked wax paper.

**Figure 12:**
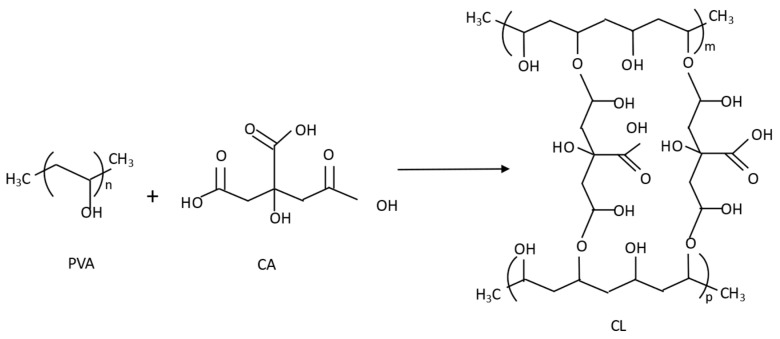
PVA and CA reaction during the cross-linking reaction.

The cross-linking between PVA and CA takes place following the reaction shown in Figure 12. As can be seen, after the cross-linking, there are still free hydroxyl groups from both PVA and CA; furthermore, there are carboxyl groups from CA as well. Turmeric added to this solution can take part in different reactions that establish turmeric retention. It is unlikely that hydrogen bonds can be formed between PVA and curcumin because neither compound has functional groups that are predisposed to form hydrogen bonds with each other, as they are formed between hydrogen atoms and another electronegative atom. However, it is possible that ester bonds are established between CA and certain ketones found in curcumin (see curcumin structure in Figure 1). Thus, we suggest that, due to the cross-linking, an ester bond is established between the free carboxyl group in the cross-linked CA and the ketones present in curcumin under acid conditions. Therefore, CA acts as an acid catalyst to bond with curcumin. Due to this reaction, the hydroxyl groups from curcumin are involved in the reaction, and consequently, the number of OH groups to be ionized (O^−^) at pH > 7 is reduced. This could be the reason for the color change associated with the halochromic effect when the nanofibers are cross-linked (CL) in comparison to the non-cross-linked ones.

Samples with thermal treatment show a cross-linked (CL) effect, as can be observed in the FTIR spectra (Figure 7). The OH stretching bands show a shift in the maximum from 3260 cm^−1^ for non-cross-linked samples (NCL) to 3306 cm^−1^ when the treated (CL) samples were analyzed. This indicates that OH groups were involved in the cross-linking reaction [41,42,43]. This behavior is also due to the hydroxyl groups from the keto form of curcumin that react with carboxyl groups from CA to create ester groups. This cross-linking between PVA and CA results in the insolubilization of the PVA nanofibers, as can be seen from the SEM results (Figure 6). The characteristic absorption of the C=O group in citric acid is observed at 1690–1750 cm^−1^. The SEM images show how a drop of alkaline liquid can dissolve the NCL samples, whereas the CL samples remain unaltered. This suggests that the thermal treatment enables the cross-linking of CA with PVA and converts soluble nanofibers into insoluble ones. This thermal procedure increases the applications of PVA as it can now be used in applications in which previously its high solubility would have prevented this use. Furthermore, CA acts by bonding the turmeric within the PVA-CA structure.

## 5. Conclusions

The use of turmeric as a natural pH indicator is well known; however, its halochromic behavior when incorporated into PVA nanofibers insolubilized by cross-linking with CA has not previously been demonstrated.

SEM microscopy showed the presence of PVA nanofibers, and their appearance, apart from some beds, was not altered by the inclusion of CA or turmeric in the solution. This means that both the turmeric and CA were completely dissolved. When the solution was filtered, no variations were observed either.

The halochromic effect was determined subjectively by visual analysis and objectively by spectrometry. Both results allow us to conclude that the non-filtered samples show higher color intensity, even though some areas of darker color can be observed in the non-filtered (NF) samples.

FTIR spectroscopy confirmed the tautomeric variation from the keto to the enol form when NaOH was added to the surface of the nanofiber veil. Furthermore, this technique demonstrated a cross-linking effect between CA and PVA.

The cross-linking treatment modifies the halochromic response but does not cancel it out altogether. This is due to the ester formed between CA and the keto form of curcumin, and we can conclude that CA plays a double role, on one hand, cross-linking with the PVA nanofibers to make them insoluble, and on the other hand, acting as an acid catalyst allowing the keto form to react with CA and bonding it to the nanofibers.

The advantage of this treatment is that the nanofibers maintain their structure even when in an alkaline environment, whereas the non-cross-linked nanofibers become completely dissolved.

This response allows insoluble PVA nanofibers to be generated and used as a non-soluble sensor, opening the door to multiple applications, including applications in the food industry and medicine.

In summary, we can highlight that CA acts as a catalyst, converting curcumin into the keto tautomeric form. This form is able to react with CA in an esterification reaction. Consequently, turmeric is entrapped by CA. The ester reaction binds some of the hydroxyl groups and thus reduces the number of O-groups, which causes the change in color. This implies a decrease in the color intensity, but the halochromic effect remains.

## Figures and Tables

**Table 1:** Characterization of the PVA solutions: polyvinyl alcohol without additives (PVA), PVA with non-filtered turmeric (PVA_NF), and PVA with filtered turmeric (PVA_F).

Reference	PVA	PVA_NF	PVA_F
Viscosity (cP)	149.13 ± 0.81	192.70 ± 4.68	129.02 ± 7.30
Conductivity (μS)	250.66 ± 13.67	1534.60 ± 13.56	1315.2 ± 12.77
Surface tension (mN/m)	65.38 ± 0.34	52.40 ± 0.82	56.78 ± 0.36
pH (pH)	5.30 ± 0.01	2.59 ± 0.01	2.54 ± 0.01

**Table 2:** Visual color test results.

Reference	Color ++++	Color +++	Color ++	Color +	No Color
Volunteers (5)	PVA_NF_120	PVA_NF_30	PVA_F_120	PVA_F_30	-

**Table 3:** CIE L*a*b* coordinates of the wax paper collector, PVA_NF_30 nanofibers, PVA_NF_120 nanofibers, PVA_F_30 nanofibers, and PVA_F_120 nanofibers.

Reference	L*	a*	b*	DL*	Da*	Db*	DE*ab	Difference
Wax paper (Standard)	94.45	0.69	−5.70	-	-	-	-	Original
PVA_NF_30	94.63	−3.72	15.07	0.18	−4.42	20.77	21.23	Yes
PVA_NF_120	96.42	−2.92	19.36	1.97	−3.61	25.06	25.40	Yes
PVA_F_30	95.38	−0.41	−0.59	0.93	−1.11	5.11	5.31	Yes
PVA_F_120	97.60	−0.48	0.62	3.15	−1.17	6.32	7.16	Yes

**Table 4:** CIE L*a*b* coordinates of the cross-linked samples: wax paper (standard); cross-linked wax paper; PVA_NF_30_CL nanofibers; PVA_NF_120_CL nanofibers; PVA_F_30_CL nanofibers; PVA_F_120_CL nanofibers.

Reference	L*	a*	b*	DL*	Da*	Db*	DE*ab	Difference
Wax paper (Standard)	94.45	0.69	−5.7	-	-	-	-	Original
Wax paper_CL	92.11	−1.24	9.37	−2.34	−1.93	15.07	15.37	Yes
PVA_NF_30_CL	94.07	−2.29	11.03	−0.38	−2.98	16.73	17	Yes
PVA_NF_120_CL	95.71	−3.83	19.82	1.26	−4.52	25.52	25.95	Yes
PVA_F_30_CL	94.21	−1.37	7.89	−0.24	−2.06	13.59	13.75	Yes
PVA_F_120_CL	96.24	−1.35	8.59	1.78	−2.04	14.3	14.55	Yes

**Table 5:** CIE L*a*b* coordinates of the wax paper collector, PVA_NF_30_H nanofibers, PVA_NF_120_H nanofibers, PVA_F_30_H nanofibers, and PVA_F_120_H nanofibers after the halochromic test.

Reference	L*	a*	b*	DL*	Da*	Db*	DE*ab	Difference
Wax paper (Standard)	94.45	0.69	−5.70	-	-	-	-	Original
PVA_NF_30_H	80.13	10.71	−9.79	−14.32	10.01	−4.09	17.94	Yes
PVA_NF_120_H	78.86	12.34	−10.13	−15.59	11.65	−4.42	19.96	Yes
PVA_F_30_H	93.77	1.34	−5.56	−0.68	0.64	0.15	0.95	Yes
PVA_F_120_H	93.88	0.32	−3.37	−0.57	−0.37	2.33	2.43	Yes

**Table 6:** CIE L*a*b* coordinates of the cross-linked samples: the wax paper collector (standard), cross-linked wax paper, PVA_NF_30_CL_H nanofibers, PVA_NF_120_CL_H nanofibers, PVA_F_30_CL_H nanofibers, and PVA_F_120_CL_H nanofibers after the halochromic test.

Reference	L*	a*	b*	DL*	Da*	Db*	DE*ab	Difference
Wax paper (Standard)	94.45	0.69	−5.7	-	-	-	-	Original
Wax paper_CL	92.11	−1.24	9.37	−2.34	−1.93	15.07	15.37	Yes
PVA_NF_30_CL_H	90.23	2.55	8.45	−4.22	1.86	14.15	14.88	Yes
PVA_NF_120_CL_H	90.06	2.95	7.87	−4.39	2.25	13.57	14.44	Yes
PVA_F_30_CL_H	91.56	−0.41	9.93	−2.89	−1.11	15.64	15.94	Yes
PVA_F_120_CL_H	92.33	−0.03	9.4	−2.12	−0.72	15.11	15.27	Yes

## Data Availability

Data are contained within the article.
